# Non-Typhoidal *Salmonella* in poultry meat and diarrhoeic patients: prevalence, antibiogram, virulotyping, molecular detection and sequencing of class I integrons in multidrug resistant strains

**DOI:** 10.1186/s13099-015-0081-1

**Published:** 2015-12-23

**Authors:** Rasha M. Gharieb, Yasmine H. Tartor, Mariam H. E. Khedr

**Affiliations:** Depatment of Zoonoses, Faculty of Veterinary Medicine, Zagazig University, 44511 Zagazig, Egypt; Department of Bacteriology, Mycology and Immunology, Faculty of Veterinary Medicine, Zagazig University, 44511 Zagazig, Egypt; Department of Veterinary Public Health, Faculty of Veterinary Medicine, Zagazig University, 44511 Zagazig, Egypt

**Keywords:** *Salmonella* spp., Zoonoses, multidrug resistance, Virulence, Class I integrons

## Abstract

**Background:**

The worldwide increase of food-borne infections with antibiotic resistant pathogens constitutes a major public health problem. Therefore, this study aimed to determine the prevalence, antibiogram, virulence genes profiles and integron characteristics of non-typhoidal *Salmonella* spp. isolated from poultry meat and diarrhoeic patients in Egypt.

**Methods:**

A total of 150 samples comprising (100 poultry meat and 50 diarrhoeic patients’ stool) were examined for the presence of *Salmonella* spp. using culture methods followed by biochemical and serological identification of the isolates. All *Salmonella* strains were tested for their susceptibility to the antibiotics using disk diffusion method and screened for the presence of virulence genes and class I integrons using PCR.

**Results:**

The overall prevalence of *Salmonella* spp. in poultry meat samples was 10 % compared to 4 % in diarrhoeic patients. All the isolates were serologically identified into *Salmonella* Typhimurium (seven isolates), *S.* Derby, *S.* Kiel, *S.* Rubislaw (one isolate, each) and untypable strains (two isolates). Antibiotic susceptibility testing showed a higher resistance of the total isolates to erythromycin and tetracycline (100 %, each), followed by amoxicillin-clavulanic acid (91.7 %), trimethoprim-sulfamethoxazole (83.3 %), streptomycin, nalidixic acid, ampicillin-sulbactam (75 %, each), gentamycin, ampicillin (66.7 %, each), chloramphenicol (58.3 %), ciprofloxacin (25 %) and ceftriaxone (16.7 %). Virulence genes profiles revealed the presence of *sop*B gene in five *Salmonella* strains isolated from poultry meat (n = 3) and humans (n = 2). Moreover, *pef*A was only identified in three isolates from poultry meat. On the other hand, *S.* Kiel and *S.* Typhimurium (one isolate, each) were harboring *hilA* and *stn* genes, respectively. Class 1 integrons were detected in all *Salmonella* spp. with variable amplicon sizes ranged from 650–3000 bp. Sequencing of these amplicons revealed the presence of gene cassettes harboring *aac(3)*-*Id*, *aadA2*, *aadA4*, *aadA7*, *sat*, *dfrA15*, *lnuF* and *estX* resistance genes. Nucleotide sequence analysis showed point mutations in the *aac(3)*-*Id* of *S.* Derby, *aadA2*, *estX*-*sat* genes of *S.* Typhimurium. Meanwhile, frame shift mutation was observed in *aad*A7 genes of *S.* Typhimurium.

**Conclusions:**

Increasing rate of antimicrobial resistance and class 1 integrons among multidrug resistant *Salmonella* spp. has prompted calls for the reduction of antimicrobial use in livestock to prevent future emergence of resistance.

## Background

Non-typhoidal *Salmonella* (NTS) is one of the major zoonotic food-borne pathogens representing an important public health problem worldwide. It can cause a variety of clinical manifestations ranging from mild gastroenteritis to bacteraemia and extra intestinal localized infections involving many organs. The global burden of NTS gastroenteritis has been estimated to be 93.8 million cases of gastroenteritis each year with 155, 000 deaths annually [1]. Food of animal origin especially poultry meat is considered to be one of the major vehicles of *Salmonella* infections in humans and has been implicated in outbreaks of human salmonellosis [2]. Antimicrobial resistance and virulence of *Salmonella* strains play a vital role in systemic infections. *Salmonella* pathogenicity is dictated by an array of factors encoded by virulence genes that assist the organism to express its virulence in the host cells and ultimately manifest in the typical symptoms of salmonellosis. Some genes are known to be involved in adhesion and invasion including; plasmid encoded fimbriae (*pefA*) and hyper invasive locus (*hilA*) [3, 4]. *Salmonella* outer proteins (sop A-E) encoded by *sop* gene and (*stn*) codes for enterotoxin productions are associated with the actual manifestation of pathogenic processes [5]. In addition to virulence factors, the emergence of antimicrobial resistance among NTS has become a public health threat. Since food of animal origin is a major source of *Salmonella* spp., it has been suggested that abundant use of antimicrobials in food animals’ production may contribute to the presence of antimicrobial resistance in these species and subsequently transferred to humans through food chain [6]. Factors contributed to the resistance and virulence of *Salmonella* may be located on chromosomes, plasmids, transposon and integrons. Integrons are DNA elements that can transfer antibiotic resistance genes among bacteria. Class I integrons are the most common type of integrons recognized among the multidrug resistant (MDR) *Salmonella* and have conserved regions (5′-CS and 3′-CS) which often contain gene cassettes [7]. These cassettes contain genes that confer resistance to antimicrobial agents including aminoglycosides, b-lactams, chloramphenicol and trimethoprim as well as genes that confer resistance to antiseptics and disinfectants [8]. The present study was undertaken to (i) trace the prevalence and antimicrobial susceptibility patterns of NTS *enterica* serotypes isolated from poultry meat and diarrhoeic patients (ii) ascertain the presence of virulence genes and class I integrons using PCR (iii) sequencing of the amplified DNA fragments of class I integrons in order to identify the resistance genes located in integron gene cassettes.

## Results and discussion

### Prevalence of *Salmonella* serovars in poultry meat and diarrhoeic patients

In the present study, the overall prevalence of *Salmonella* spp. in the total examined poultry meat samples was 10 % (Table 1). This result was compatible with previous studies in Nepal [9]. Moreover, our results regarding the prevalence of *Salmonella* spp. in broiler chicken meat (14 %) substantiate the findings of others in Canada [10].

**Table 1:** Frequency distribution of *Salmonella* serovars in poultry meat and diarrhoeic patients

Source	Poultry meat			Diarrhoeic patients (n = 50)	Total (150)
	Chicken (n = 50)	Duck (n = 50)	Total (n = 100)		
	No. (%)	No. (%)	No. (%)	No. (%)	No. (%)
Serotype					
*S.* Typhimurium (O1,4,[5],12 H_1_: i H_2_: 1,2)	5 (10)	0 (0)	5 (5)	2 (4)	7 (4.7)
*S.* Rubislaw (O11 H_1_: r H2: e,n,x)	1 (2)	0 (0)	1 (1)	0 (0)	1(0.7)
*S.* Kiel (O1,2,12 H_1_: g, p H_2_:–)	1 (2)	0 (0)	1 (1)	0 (0)	1 (0.7)
*S.* Derby (O1,4,[5],12 H_1_: f,g H2: [1,2])	0 (0)	1 (2)	1 (1)	0 (0)	1 (0.7)
Untypable	0 (0)	2 (4)	2 (2)	0 (0)	2 (1.3)
Total	7 (14)	3 (6)	10 (10)	2 (4)	12 (8)

Serotyping of *Salmonella* spp. isolated from poultry meat in this study revealed the predominance of *S.* Typhimurium in chicken meat and this was in concordance with previous studies from India [11]. Additionally, other serovars such as *S.* Rubislaw, *S.* Kiel (chicken meat) and *S.* Derby (duck meat) were identified.

It was obvious from Table 1 that 4 % (2 out of 50) of stool samples from diarrhoeic patients were positive for *Salmonella* spp. and *S.* Typhimurium was the only serotype identified. Nearly similar prevalence in diarrhoeic patients was reported in India [12]. The isolation of *S.* Derby from poultry meat and humans has been previously recorded in Netherlands [13]. Moreover, *S.* Rubislaw was isolated from chicken meat in Senegal and diarrhoeic children in Gambia [14] and this confirms the zoonotic importance of the former serotypes.

### Antibiotic susceptibility testing

All *Salmonella* isolates were tested for their susceptibility towards antimicrobial drugs (Table 2). The results highlighted the higher resistance of the isolates to erythromycin and tetracycline (100 %, each), followed by amoxicillin-clavulanic acid (91.7 %), trimethoprim-sulfamethoxazole (83.3 %), streptomycin, nalidixic acid, ampicillin-sulbactam (75 %, each), gentamycin, ampicillin (66.7 %, each), chloramphenicol (58.3 %), ciprofloxacin (25 %) and ceftriaxone (16.7 %). This isn’t surprising because these antibiotics are cheap, easily affordable and commonly used in humans and poultry without prescription. In poultry, these drugs are used either for therapeutic purposes or as growth promoters added to the feed leading to the development of resistance in the enteric bacterial flora of poultry. Subsequently the pathogenic bacteria such as *Salmonella* may acquire resistance from this enteric flora and transfer this resistance to human’s strains through food chain leading to the emergence of MDR *Salmonella* strains that constitute a public health risk and potentially affect the efficacy of drug treatment in humans. It was obvious from Table 2 that 100 % of *Salmonella* spp. isolated from poultry meat were resistant to each of erythromycin and tetracycline, 9 (90 %) were resistant to amoxicillin-clavulanic acid, 8 (80 %) showing resistance to trimethoprim-sulfamethoxazole, 7 (70 %) exhibiting resistance to each of streptomycin, ampicillin-sulbactam, nalidixic acid and gentamycin. Moreover, the lower resistance rates to chloramphenicol (50 %), ciprofloxacin (30 %) and ceftriaxone (10 %) were observed. Nearly similar resistance rate to ampicillin, streptomycin, nalidixic acid, tetracycline and trimethoprim-sulfamethoxazole was recorded in *Salmonella* spp. isolated from chicken meat in Pakistan [15]. On the contrary, the lower resistance rate to ceftriaxone and ciprofloxacin corroborates with others in India [11].

**Table 2:** Antibiogram of *Salmonella* serovars isolated from poultry meat and diarrhoeic patients

Sources/serovars	Antibiotic (concentration µg)											
	A (10)	E (15)	S (10)	Cx (5)	T (30)	Co (30)	As (20)	Sxt (25)	C (30)	Na (30)	G (10)	Amc (30)
Chicken meat (7)	4 (57.1)	7 (100)	5 (71.4)	2 (28.6)	7 (100)	1 (14.3)	5 (71.4)	6 (85.7)	4 (57.1)	5 (71.4)	6 (85.7)	6 (85.7)
*S.* Typhimurium (5)	2 (20)	5 (100)	4 (80)	2 (40)	5 (100)	1 (20)	4 (80)	5 (100)	3 (60)	4 (80)	5 (100)	4 (80)
*S.* Kiel (1)	1 (100)	1 (100)	0	0	1 (100)	0	0	0	0	0	0	1 (100)
*S.* Rubislaw (1)	1 (100)	1 (100)	1 (100)	0	1 (100)	0	1 (100)	1 (100)	1 (100)	1 (100)	1 (100)	1 (100)
Duck meat (3)	2 (66.7)	3 (100)	2 (66.7)	1 (33.3)	3 (100)	0	2 (66.7)	2 (66.7)	1 (33.3)	2 (66.7)	1 (33.3)	3 (100)
*S.* Derby(1)	0	1 (100)	1 (100)	0	1 (100)	0	0	0	0	0	0	1 (100)
Untypable(2)	2 (100)	2 (100)	1 (50)	1 (50)	2 (100)	0	2 (100)	2 (100)	1 (50)	2 (100)	1 (50)	2 (100)
Total (10)	6 (60)	10 (100)	7 (70)	3 (30)	10 (100)	1 (10)	7 (70)	8 (80)	5 (50)	7 (70)	7 (70)	9 (90)
Human (2) *S.* Typhimurium	2 (100)	2 (100)	2 (100)	0	2 (100)	1 (50)	2 (100)	2 (100)	2 (100)	2 (100)	1 (50)	2 (100)
Total sensitive (%)	2 (16.7)	0	2 (16.7)	4 (33.3)	0	4 (33.3)	1 (8.3)	2 (16.7)	2 (16.7)	0	2 (16.7)	0
Total intermediate (%)	2 (16.7)	0	1 (8.3)	5 (41.7)	0	6 (50)	2 (16.7)	0	3 (25)	3 (25)	2 (16.7)	1 (8.3)
Total resistant (%)	8 (66.7)	12 (100)	9 (75)	3 (25)	12 (100)	2 (16.7)	9 (75)	10 (83.3)	7 (58.3)	9 (75)	8 (66.7)	11 (91.7)

*A* Ampicillin, *E* Erythromycin, *S* Streptomycin, *Cx* Ciprofloxacin, *T* Tetracycline, *Co* Ceftriaxone, *As* Ampicillin-Sulbactam, *Sxt* Trimethoprim-Sulfamethoxazole, *C* Chloramphenicol, *Na* Nalidixic acid, *G* Gentmycin, *Amc* Amoxicillin-Clavulanic acid

Table 2 also showed that all *Salmonella* isolates of human origin were resistant to ampicillin, erythromycin, streptomycin, tetracycline, ampicillin-sulbactam, trimethoprim- sulfamethoxazole, chloramphenicol, nalidixic acid and amoxicillin-clavulanic acid. Conversely, the resistance to ceftriaxone and gentamycin was found in 50 % of the isolates. On the other hand, none of the isolates was resistant to ciprofloxacin. Consistent with our results, a higher resistance rate (100 %) to ampicillin, chloramphenicol, streptomycin, trimethoprim-sulfamethoxazole and tetracyclines was recorded in *S.* Typhimurium strains isolated from diarrhoeic patients in Kenya [16]. Another worrisome situation in this study is that, 50 % of *Salmonella* isolates from diarrhoeic patients were resistant to ceftriaxone and gentamycin. This is of particular concern because the extended spectrum cephalosporins such as ceftriaxone are the antibiotics of choice for treatment of invasive salmonellosis in children and the emergence of resistance toward these drugs could be attributed to inappropriate use of them in treatment of *Salmonella* infections in humans. The higher susceptibility of *S*. Typhimurium isolated from human source to ciprofloxacin (100 %) in this study may be due to discriminate use of this drug because it is relatively expensive, not easily affordable to all people, not sold in private pharmacies without prescription. This result corroborates the findings of other investigators in Kenya [16]. Therefore, ciprofloxacin still to have a high potency against NTS strains in humans and continue to be the most successful drug used for treatment of septicaemic salmonellosis in adult humans. The increased resistance of *S.* Typhimurium isolated from humans to quinolones (nalidixic acid) in this study (100 %) is a matter of concern and could be attributed to indiscriminate use of these antibiotics by human patients. Moreover, the presence of these drug residues in food of animal origin may results in increased resistance among human’s isolates.

Table 3 verified that all *Salmonella* spp. isolated from poultry and humans were MDR, exhibiting resistance to at least four or more antibiotics displayed by ten resistance patterns.

**Table 3:** Resistance pattern, virulence genes and class I integron profiles among multidrug resistant *Salmonella* serotypes in this study

Strain no.	Serovar (source of isolate)	Resistance pattern	Virulence genes				Integron amplicon size (bp)	Integron profile (IP)	Genes in the cassettes	Accession numbers
			*Pef*A	*hil*A	*Sop*B	Stn				
1	S. Derby (duck)	ST E Amc	−	−	−	−	937 1600	I	*aac (3)-Idaac (3)-Id, aadA7*	KT581257 KT427378
2	S. Kiel (chicken)	A T E Amc	−	+	−	−	937	II		
3	Untypable (duck)	A C S Sxt T E Cx As Na G Amc	+	−	+	−	1500	III	*aac (3)-Id, aadA7*	KT581256
4	S. Rubislaw (chicken)	A C S Sxt T E As Na G Amc	+	−	−	−	1500	III	–	
5	S. Typhimurium (chicken)	A C S Sxt T E As Na G Amc	−	−	−	−	1100 1500	IV	– *estX-sat*	KT581255
6	S. Typhimurium (chicken)	S Sxt T E Cx Co Na G	−	−	+	−	650 1550	V	*sat* *ac (3)-Id, aadA7at*	KT449570 KT598359
7	Untypable (duck)	A Sxt T E As Na Amc	−	−	+	−	1600 3000	VI	–	
8	S. Typhimurium (human)	A C S Sxt T E Cro As Na G Amc	−	−	+	−	1000	VII	–	
9	S. Typhimurium (human)	A C S Sxt T E As Na Amc	−	−	+	+	650 750 1600	VIII	*adA4* *adfrA15*	KT581253 KT449571
10	S. Typhimurium (chicken)	S Sxt T E As G Amc	−	−	−	−	1900	IX		
11	S. Typhimurium (chicken)	A C S Sxt T E As Na G Amc	−	−	−	−	1900	IX	*aadA2, lnuF*	KT449569
12	S. Typhimurium (chicken)	C Sxt T E Cx As Na G Amc	+	−	−	−	1550	X	*aac (3)-Id, aadA7*	KT581254

### Molecular detection of *Salmonella* virulence genes (virulotyping)

**Fig. 1:**
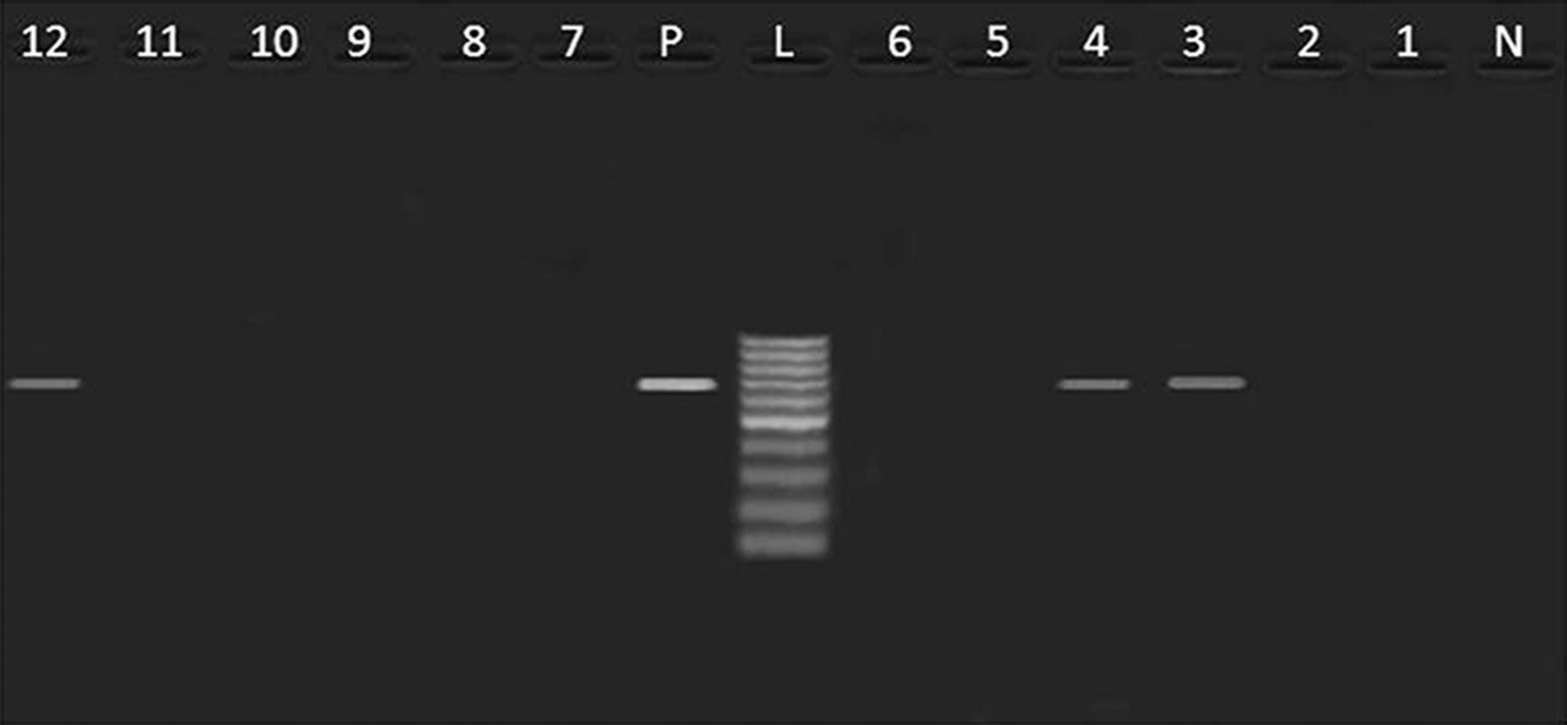
*Agarose gel electrophoresis* showing an amplification of *pefA* gene (700 bp) in *Salmonella* isolates from poultry meat and humans. Lane N: negative control (*pef*A^−^ *Salmonella* strain), lane L: DNA ladder (100 bp), lane P: positive control (*pef*A^+^ *Salmonella* strain), lane 1: *pef*A^−^ (*S.* Derby, duck), lane 2: *pef*A^−^ (*S.* Kiel, chicken), lane3: *pef*A^+^ (untypable, duck), lane 4: *pef*A^+^ (*S.* Rubislaw, chicken), lanes 5,6, 10, 11: *pef*A^−^ (*S.* Typhimurium, chicken), lane 7: *pef*A^−^ (untypable, duck), lanes 8,9: *pef*A^−^ (*S.* Typhimurium, humans), lane12: *pef*A^+^ (*S.* Typhimurium, chicken)

**Fig. 2:**
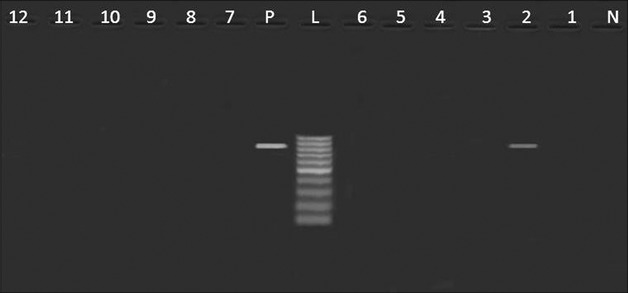
*Agarose gel electrophoresis* showing an amplification of *hil*A gene (854 bp) in *Salmonella* isolates from poultry meat and humans. Lane N: negative control (*hilA* ^−^ *Salmonella* strain), lane L: DNA ladder (100 bp), lane P: Positive control (*hil*A^+^ *Salmonella* strain), lane 1: *hilA* ^−^ (*S.* Derby, duck), lane 2: *hil*A^+^ (*S.* Kiel, chicken), lane 3,7: *hil*A^−^ (untypable, duck), lane 4: *hil*A^−^ (*S.* Rubislaw, chicken), lanes 5, 6, 10, 11,12: *hil*A^−^ (*S.* Typhimurium, chicken), lanes 8,9: *hil*A^−^ (*S.* Typhimurium, humans)

**Fig. 3:**
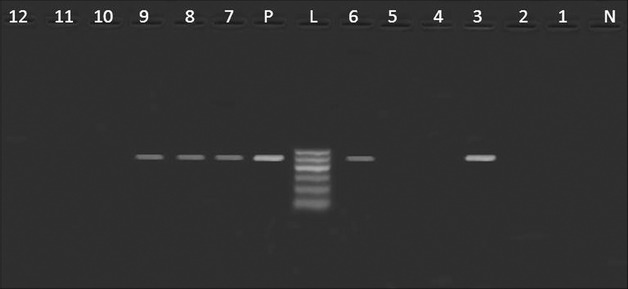
*Agarose gel electrophoresis* showing an amplification of *sop*B gene (517 bp) in *Salmonella* isolates from poultry meat and humans. Lane N: negative control (*sop*B^−^ *Salmonella* strain), lane L: DNA ladder (100 bp), lane P: Positive control (*sop*B^+^ *Salmonella* strain), lane 1: *sop*B^−^ (*S.*Derby, duck), lane 2: *sop*B^−^ (*S.* Kiel, chicken), lanes 3, 7: *sop*B^+^ (untypable, duck), lane 4: *sop*B^−^ (*S.* Rubislaw, chicken), lanes 5: *sop*B^−^ (*S.* Typhimurium, chicken), lane 6: *sop*B^+^ (*S.* Typhimurium, chicken), lanes 8,9: *sop*B^+^ (*S.* Typhimurium, humans), lane 10, 11, 12: *sop*B^−^ (*S.* Typhimurium,chicken)

Table 3 revealed the presence of *pef*A gene among three *Salmonella* strains isolated from poultry meat, belonged to *S.* Rubislaw, *S.* Typhimurium and untypable strain (one isolate, each) (Fig. 1). Moreover, *hil*A *gene* was only identified in one isolate of *S.* Kiel (Fig. 2). Furthermore, three *Salmonella* isolates from poultry meat comprising untypable strains (two isolates) and *S.* Typhimurium (one isolate) were harboring *sop*B gene (Fig. 3). The detection of *pef*A gene in *S.* Rubislaw in Gambia and Senegal [14] and *S.* Typhimurium in India [4] was previously reported. On the other hand, *hil*A gene was identified in *Salmonella* serotypes isolated from chickens in Iran [17].

**Fig. 4:**
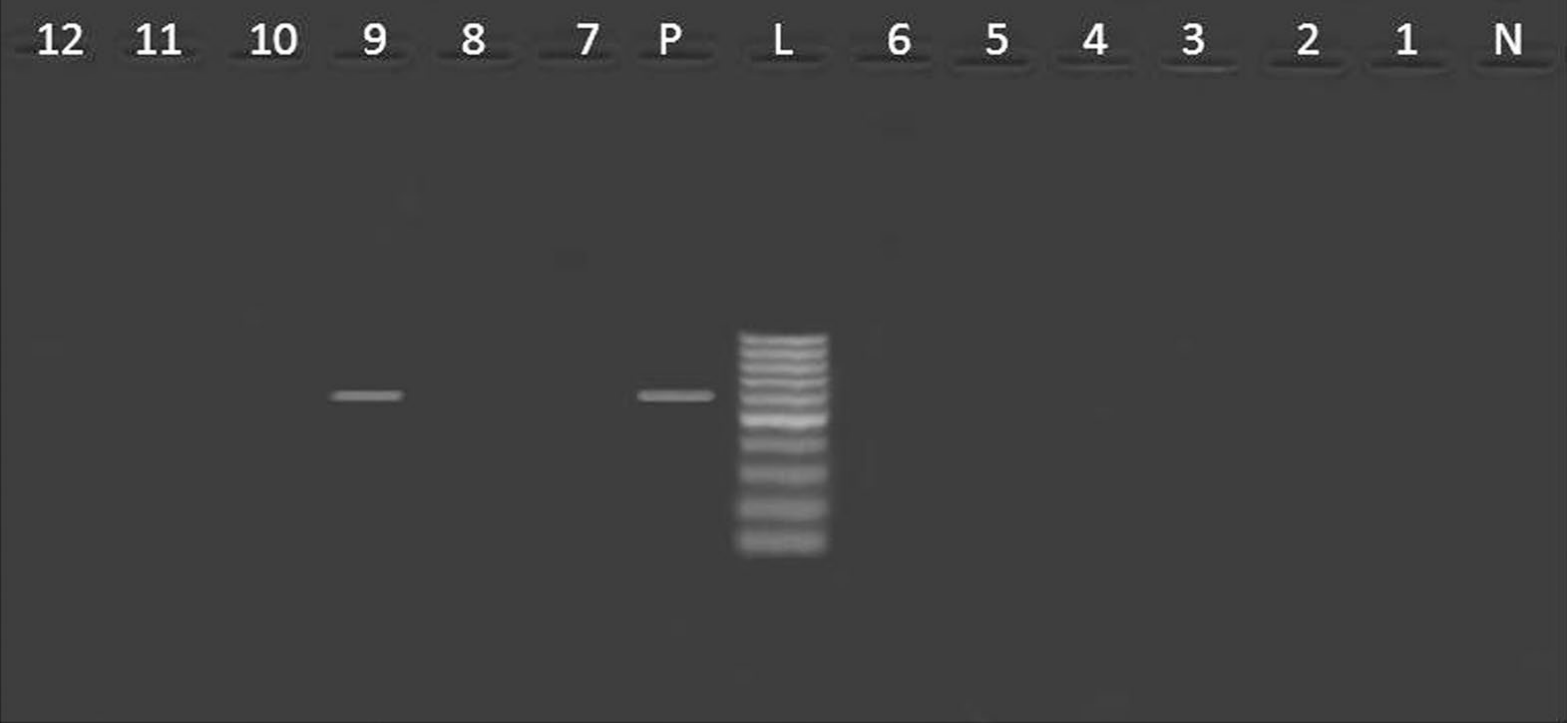
*Agarose gel electrophoresis* showing an amplification of *stn* gene (617 bp) in *Salmonella* isolates from poultry meat and humans. Lane N: negative control (*stn* ^−^ *Salmonella* strain), lane L: DNA ladder (100 bp), lane P: Positive control (*stn* ^+^ *Salmonella* strain), lane 1: *stn* ^−^ (*S.* Derby, duck), lane 2: *stn* ^−^ (*S.* Kiel, chicken), lanes 3,7: *stn* ^−^ (untypable, duck), lane 4: *stn* ^−^ (*S.* Rubislaw, chicken), lanes 5,6,10,11,12: *stn* ^−^ (*S.* Typhimurium, chicken), lane 8: *stn* ^−^ (*S.* Typhimurium, humans), lane 9: *stn* ^+^ (*S.* Typhimurium, humans)

It was obvious from Table 3 that all *S.* Typhimurium isolates from humans were harboring *sop*B compared to one isolate had *stn* gene (Figs. 3, 4). Consistent with our findings, *sop*B gene was previously identified in *S.* Typhimurium isolated from diarrhoeic patients and birds in India [18]. Meanwhile, *stn* gene was detected in *S.* Typhimurium isolated from humans in India [4].

### Molecular detection and sequencing of class 1 integrons

**Fig. 5:**
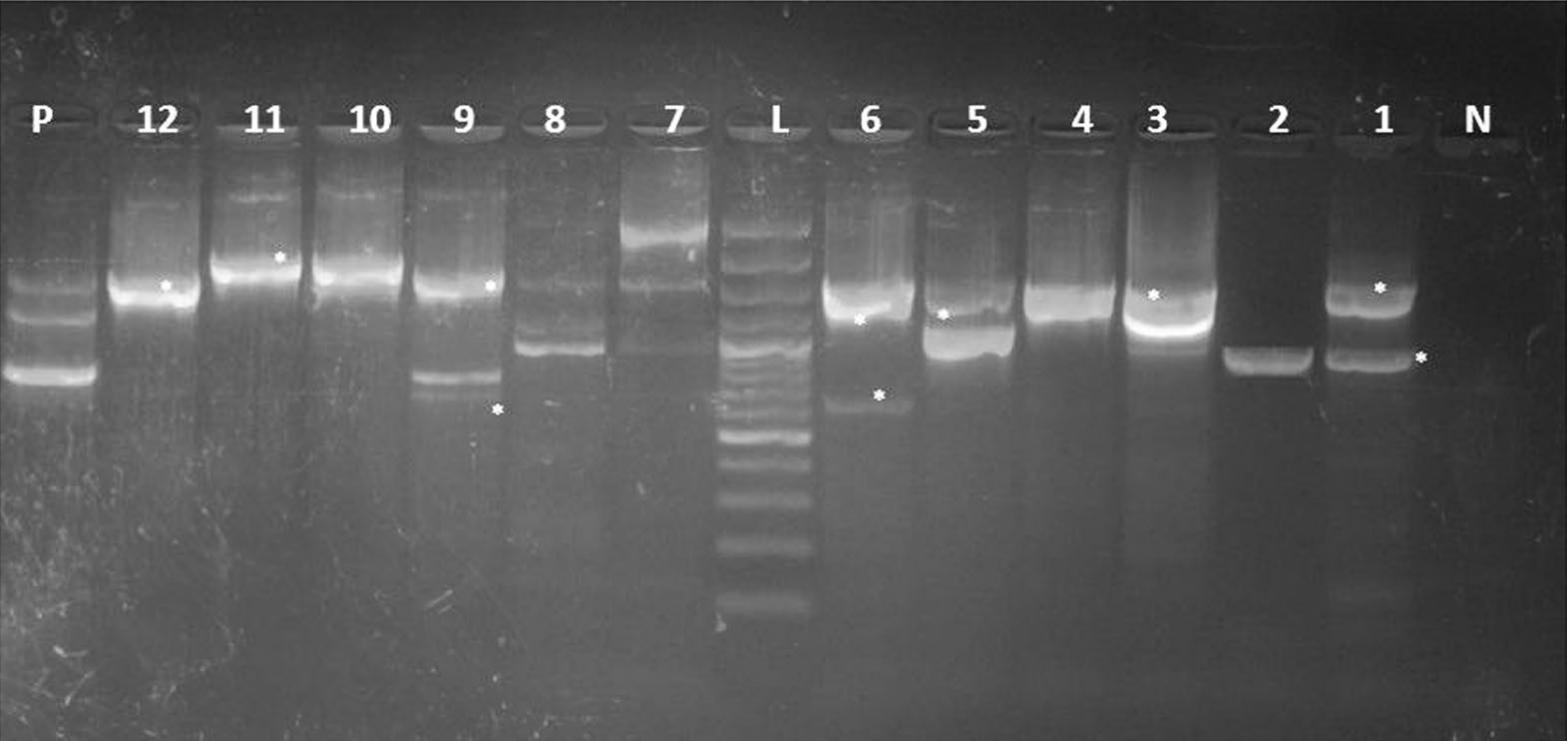
Amplification of conserved regions (5′-CS and 3′-CS) of class 1 integron and integron profiles (IPs) detected in *Salmonella* isolates from poultry meat and humans. *Asterisks* represent the bands selected for sequencing. Lane N: negative control, lane L: DNA ladder (100 bp), lane P: Positive control, lane 1: IP I *(S.* Derby, duck), lane 2: IP II (*S.* Kiel, chicken), lanes 3,4: IP III (untypable, duck & *S.* Rubislaw, chicken), lanes 5, 6,10,11, 12: IP IV, V, IX, X (*S.* Typhimurium, chicken), lanes 8, 9: IPs VII, VIII (*S.* Typhimurium, humans)

A notable feature in this study is that all *Salmonella* strains isolated from poultry meat and humans were MDR and harboring class I integrons that are chromosomally located (Fig. 5). Ten different profiles of class I integrons with variable amplicon sizes ranged from 650–3000 bp were observed (Table 3). Similarily, class I integrons were detected in 97 % of *S.* Typhimurium isolated from Norwegian diarrhoeic patients [19]. On the contrary, a lower detection rate (9 %) was recorded in *Salmonella* spp. isolated from poultry meat [20]. Sequencing of the variable amplicons of class I integrons denoted by asterisks (Fig. 5) revealed the presence of gene cassettes containing aminoglycoside acetyltransferase [*aac(3)*-*Id* or *aacCA5*] gene which confers resistance against gentamycin, aminoglycoside adenyltransferase (*aadA2*, *aadA4*, *aadA7*) genes that confer resistance to streptomycin and spectinomycin, streptothricin acetyltransferase (*sat*) encoding resistance against streptothricin, dihydrofolate reductase type 15 (*dfrA15*) that confers resistance to trimethoprim, *lnuF* gene that codes for lincosamides resistance (lincomycin and clindamycin) and putative esterase (*estX*). Interestingly, the *aac (3)*-*Id* plus *aadA7* were the most predominant resistance genes identified in class I integron gene cassettes of 1600 bp (*S.* Derby, duck), 1500 bp (untypable strain, duck) and 1550 bp (*S.* Typhimurium, chicken). The same cassettes harboring *aac (3)*-*Id* and *aadA7* genes were previously identified in class I integron of *S.* Haifa (GenBank accession no. AY563051) [21], *S.* Newport (AY458224) [22] and *S.* Kentucky (AY463797) [23]. Despite the presence of these genes, some strains such as *S.* Derby was susceptible to gentamycin and *S.* Typhimurium strains (chicken) were sensitive to streptomycin and this could be attributed to point mutations in the *aac(3)*-*Id* gene of *S.* Derby [24] and frame shift mutation in *S.* Typhimurium [25, 26]. Nucleotide sequence analysis revealed point mutations in the *aac(3)*-*Id* gene of *S.* Derby leading to the amino acid changes at codon 36 (glutamine CAG→proline CCG), codon 166,172 (arginine AGA→threonine ACA), codon 187 (arginine CGA→CTA leucine) and codon 189 (cysteine TGC→arginine CGC).C-to-T transition at position 208 of the deduced polypeptide resulted in a leucine to phenylalanine substitution and A to C transversion at codon 214 resulted in asparagine to histidine substitution. Silent mutation was recorded only at nucleotide 138 and didn’t result in amino acid substitution (CCA→CCG, both are proline). Moreover, sequence analysis of *aadA7* gene showed frame shift mutation due to additional cytosine at position 969 of the deposited sequence of *S.* Typhimurium from chicken.

In the present study, only one isolate of *S.* Typhimurium from human was found to harbor class I integrons of 650, 750 and 1600 bp. Sequence analysis of 650 bp revealed the presence of gene cassette containing *aadA4* which showed 100 % identity with *aadA4* previously identified in class I integron of *S.* Newport (CP006631) and plasmid of *S.* Indiana (ref|NG_041636). In addition, the 1600 bp integron carried gene cassette containing *dfrA15* gene that showed 100 % amino acid and nucleotide homology with *dfrA15* gene previously reported in *S.enterica* strain (KM823524).

The *sat* gene detected in the gene cassette of class I integron (650 bp) of *S.* Typhimurium in this study was previously identified in *S.* Kedougou (DQ284538) [27] and plasmid of *S.* Choleraesuis (AY509004) [28]. Moreover, the 1500 bp amplicon identified in *S.* Typhimurium contained *estX*-*sat* gene cassette that is identical to *est*X gene previously identified in *S.* Typhimurium (EF051039), *sat* gene of *S.* Choleraesuis plasmid (ref|NG_036624) and SC-B67 strain (AY509004). Point mutations were observed in *estX*-*sat* gene cassette in this study compared to the classical *estX* and *sat* genes (99 % nucleotide identity); these genes displayed both missense and silent mutations. The nucleotide number 763 of *estX* was exchanged from T to C resulting in exchange of amino acid (phenylalanine) into leucine. Furthermore, at codon 288 (histidine TAC→tyrosine CAC) and 290 (threonine ACA→alanine GCA). Silent mutations were recorded at nucleotides 894 (GTC→GTA, alanine), 903 (TAT→TAC, tyrosine) and 927 (GTC→GTA, both are valine). While in *sat* gene, silent mutations were detected at codon 418 (ACT→ACC, tyrosine), 428 (GTC→GTA, valine), 451 and 453 (GGC→GGT, GGA→GGG both are glycine). Amino acids replacement was recorded in *sat* gene at codon 459 (lysine AAA→glutamic acid GAA), 462 (proline CCG→leucine CTG) and 465 (isoleucine ATC→threonine ACC). Nonsense mutation was predicted at nucleotide 1418 (serine TCA→stop codon TAA). Antimicrobial drug resistance can occur by point mutations in the bacterial genome [29] or through mobile genetic elements called integrons which are able to disseminate the antimicrobial resistance among the enterobacteriaceae by horizontal transfer [30].

In fact, this study reports for the first time the presence of *aadA2* plus *lnuF* genes within the same integron gene cassette of 1900 bp in *S.typhimurium* from chicken. The nucleotide sequence of *aadA2* and *lnuF* genes, respectively showed 99 and 100 % identity with *aadA2* and *lnuF* genes previously reported in *S. enterica* serovar Stanley (EU118119). Moreover, the *aadA2* gene had 99 % amino acid and nucleotide homology with *aadA2* previously identified in *S.* Typhimurium isolated from food-animals and humans in Mexico (FJ460233) [31]. On the other hand, the 1900 bp of class I integron was found to harbor *aadA2* plus *dfrA12* in *S. enterica* serovar Typhimurium isolated from food-animals, chicken and humans in Malaysia [32].

**Fig. 6:**
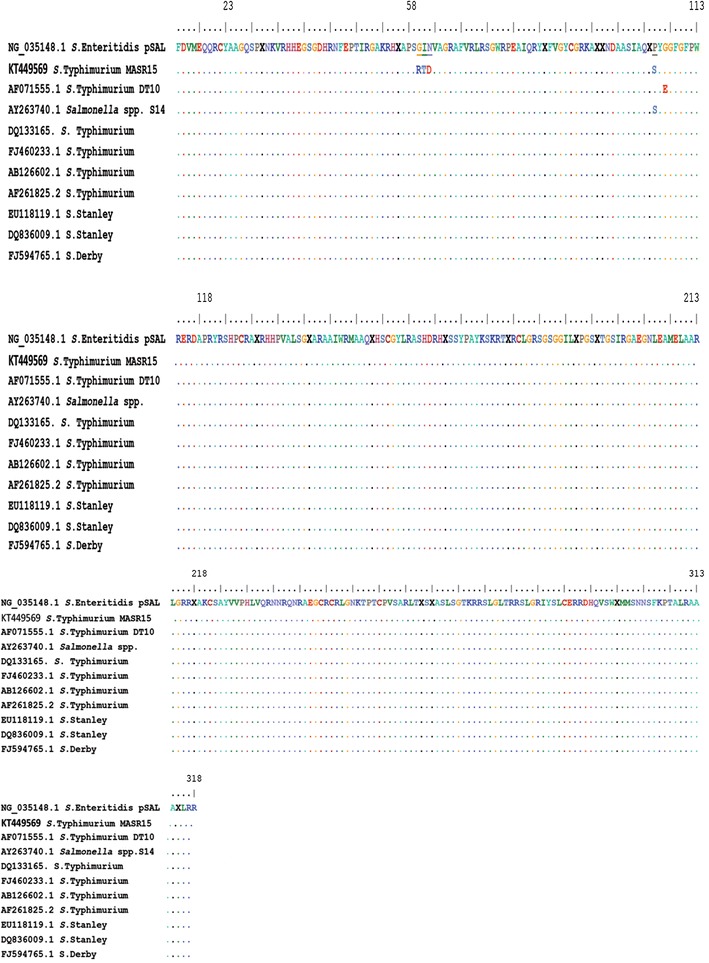
Amino acid sequence similarities for *S.* Typhimurium *aadA2* gene of the strain under study (*S.* Typhimurium strain MASR STKT449569) and the reference strains. *Dots* indicate amino acid positions that are identical to the corresponding *S.* Typhimurium *aadA2* sequence. The glycine 60, isoleucine 61, asparagine 62 and proline 105 in which mutations occur are indicated by the *solid bars*

The nucleotide sequence of *aadA2* showed four base differences from other *aadA2* in GenBank reported cassettes and this caused point mutation which are responsible for an alteration in the codon 60 (glycine GGA→arginine CGA), codon 61(isoleucine ATC→threonine ACC), codon 62 (asparagine AAC→aspartic acid GAC) and codon 105 (proline CCT→TCT serine) (Fig. 6). Similarly, point mutation in the *aadA2* gene of NTS *enterica* isolates was reported [29].

## Conclusions

The higher resistance rates displayed by *Salmonella* isolates in this study to the antibiotics and the detection of class 1 integron gene cassettes harboring resistance genes among MDR strains shows the potential of integrons to carry and spread resistance genes to other *S. enterica* isolates or to other bacteria. Therefore, a more prudent use of antibiotics in both humans and animals is required.

## Methods

### Ethical approval

This study was approved by Zagazig University, Egypt ethical board (Protocol No. 10122). Permission to collect stool samples was obtained from the administrators of Al-Ahrar General hospital.

### Specimen collection

The present work was conducted during the period from March, 2014 to February, 2015. A total of one hundred fresh poultry carcasses comprising broiler chicken and ducks (50, each) were randomly purchased from poultry retail outlets at Zagazig City, Egypt. In addition, fifty stool samples were collected from the patients attending the outpatients’ clinic of Al-Ahrar General Hospital. The inclusion criteria were restricted to the patients who suffered from diarrhoea, fever and gave informed written consent to participate in the study. Moreover, patients received antibiotic treatments were excluded. A swab was obtained from each freshly passed stool sample, labeled, ice packed and transported to the Bacteriology Laboratory, Faculty of Veterinary Medicine, Zagazig University, Egypt within 24 h for bacteriological analysis.

### Isolation and identification of *Salmonella* spp

*Salmonella* isolation was carried out according to the standard methods recommended by ISO 6579 [33]. Twenty-five grams of poultry meat were excised from each sample and minced with 225 ml of buffered peptone water (BPW) enrichment broth. Also, each stool swab was inserted in sterile tube containing 9 ml BPW. All samples were incubated at 37 °C for 24 h. Aliquots (0.1 ml) of the pre-enriched culture was inoculated into 10 ml of Rappaport Vasiliadis (RV) enrichment broth and incubated at 42 °C for 24 h. A loopful of the enriched culture was streaked on Xylose-Lysine-Deoxycholate (XLD) agar plates (Oxoid, Basingstoke, UK) and incubated at 37 °C for 24 h. Suspected colonies with typical *Salmonella* morphology were confirmed biochemically by indole, citrate utilization, urease, triple sugar iron (TSI) and lysine iron agar (LIA) tests. All biochemically confirmed *Salmonella* isolates were serologically identified on the basis of somatic (O) and flagellar (H) antigens by slide agglutination using commercial antisera (SISIN, Berlin) following Kauffman-White scheme [34] in collaboration with Serology Unit, Animal Health Research Institute, Dokki, Egypt.

### Antimicrobial susceptibility testing (antibiogram)

Susceptibility of *Salmonella* isolates to various routine antimicrobial drugs was tested by the standard disc diffusion technique [35] using Mueller–Hinton agar and commercial antibiotic discs (Oxoid, UK). The antibiotics used were ampicillin (10 μg), erythromycin (15 μg), streptomycin (10 μg), ciprofloxacin (5 μg), tetracycline (30 μg), ceftriaxone (30 μg), ampicillin-sulbactam (20 μg), trimethoprim-sulfamethoxazole (25 μg), chloramphenicol (30 μg), nalidixic acid (30 μg), gentamicin (10 μg), amoxicillin-clavulanic acid (30 μg). The inhibition zones in mm were measured and scored as sensitive, intermediate and resistant categories in accordance with the critical breakpoints recommended by the Clinical and Laboratory Standards Institute [36].

### Molecular detection of *Salmonella* virulence genes using uniplex PCR

A total of twelve *Salmonella* isolates were screened for the presence of virulence genes using PCR. These genes including; plasmid encoded fimbriae (*pef*A), hyper invasive locus (*hil*A), *Salmonella* outer protein (*sop*B) and *Salmonella* enterotoxin gene (*stn*). Briefly, DNA was extracted from the overnight bacterial culture using ABIO pure Genomic DNA extraction kit with modifications from the manufacturer’s recommendation. DNA amplification was carried out using specific primers supplied from Metabion, Germany (Table 4). Primers were utilized in a 25 µl reaction containing 12.5 µl of Emerald Amp Max PCR Master Mix (Takara, Japan), one µl of each primer (20 pmol concentration), 4.5 µl of nuclease free water, and 6 µl of DNA template. The reaction was performed in a T3 Biometra thermal cycler. Positive and negative controls were included in each reaction. Aliquot of each amplicon along with a 100 bp molecular weight DNA ladder (Fermentas, USA) were separated by electrophoresis on 1.5 % agarose gel (Applichem, Germany, GmbH) stained with 0.5 μg/ml ethidium bromide (Sigma, USA) in 1 × TBE buffer on a mini slab horizontal electrophoresis unit (Bio-Rad, USA) at 100 V for 30 min. The gel was photographed by a gel documentation system (Alpha Innotech, Biometra) and the data was analyzed through computer software.

**Table 4:** Primers’ sequences and amplification’s conditions used for detection of *Salmonella* virulence genes

Target gene	Primers’ sequences 5′–3′	Amplified segment (bp)	Primary denaturation	Amplification (35 cycles)			Final extension	References
				Secondary denaturation	Annealing	Extension		
*pef*A	TGTTTCCGGGCTTGTGCT	700	94 °C 10 min	94 °C 45 s	55 °C 45 s	72 °C 45 s	72 °C 10 min	[4]
	CAGGGCATTTGCTGATTCTTCC							
*hil*A	CGGAAGCTTATTTGCGCCATGCT GAGGTAG	854	94 °C 10 min	94 °C 45 s	60 °C 45 s	72 °C 45 s	72 °C 10 min	[37]
	GCATGGATCCCCGCCGGCGAGAT TGTG							
*sop*B	TCAGAAGRCGTCTAACCACTC	517	94 °C 5 min	94 °C 30 s	58 °C 30 s	72 °C 30 s	72 °C 7 min	[38]
	TACCGTCCTCATGCACACTC							
*stn*	TTGTGTCGCTATCACTGGCAACC	617	94 °C 10 min	94 °C 45 s	59 °C 45 s	72 °C 45 s	72 °C 10 min	[4]
	ATTCGTAACCCGCTCTCGTCC							

### Molecular detection and sequencing of class 1 integrons

All MDR *Salmonella* isolates (n = 12) were tested for the presence of conserved regions (5′-CS and 3′-CS) of class Ι integrons using specific primers with the following sequences: 5′ CS-GGC ATC CAA GCA GCA AG and 3′CS- AAG CAG ACT TGA CCT GA [30]. Chromosomal DNA extraction, amplification and gel analysis were performed as mentioned before except to amplify the DNA in the thermal cycler we used a three-step profile (cycling conditions): 1 min of denaturation at 94 °C, 1 min of annealing at 55 °C, and 5 min of extension at 72 °C for a total of 35 cycles. Five seconds were added to the extension time at each cycle. Amplified products with variable sizes were purified using QIA quick PCR Product extraction kit (Qiagen, Valencia). Each purified amplicon was sequenced in both forward and reverse directions using the amplification primers. DNA sequences were obtained by Applied Biosystems 3130 genetic analyzer (HITACHI, Japan).Nucleotide sequence analysis was compared with published GenBank DNA sequences using NCBI BLAST program (http://www.ncbi.nlm.nih.gov/BLAST/) and alignment was performed usingMEGA6 program.

### Nucleotide sequence accession numbers

Nucleotide sequences from this study were deposited into the GenBank under accession numbers [KT427378, KT449569, KT449570, KT449571, KT581253, KT581254, KT581255, KT581256, KT581257 and KT598359].
